# Retraction:

**DOI:** 10.1002/ctm2.1077

**Published:** 2022-10-25

Weixin Yan, Lingpeng Xie, Yanmeng Bi, Ting Zeng, Di Zhao, Yuqi Lai, Tingting Gao, Xuegang Sun, Yafei Shi, Zhaoyang Dong, Ge Wen, Lei Gao, Zhiping Lv. Combined rs‐fMRI study on brain functional imaging and mechanism of RAGE‐DAMPs of depression: Evidence from MDD patients to chronic stress‐induced depression models in cynomolgus monkeys and mice. *Clinical and Translational Medicine*, 2021:e541. The above article, published online on 12 October 2021 in Wiley Online Library (wileyonlinelibrary.com), has been retracted by agreement between the authors, the journal Editor in Chief Xiangdong Wang, and John Wiley & Sons Australia, Ltd. The retraction has been agreed due to the shortcomings of the cynomolgus monkey depression model. The isolated conditions applied, and the flawed behavioural classification method and the insufficient experimental duration of the animal model render the conclusions of the article inaccurate.

## References

[ctm21077-bib-0001] Yan W , Xie L , Bi Y , et al. Combined rs‐fMRI study on brain functional imaging and mechanism of RAGE‐DAMPs of depression: Evidence from MDD patients to chronic stress‐induced depression models in cynomolgus monkeys and mice. Clin Transl Med. 2021;10:e541. 10.1002/ctm2.541 PMC8506644 34709765

